# "Tree-Day" at Manchester Children's Hospital

**Published:** 1913-01-11

**Authors:** 


### "TREE-DAY" AT MANCHESTER CHILDREN'S HOSPITAL.

"Tree-Day" at the Manchester Children's Hospital, Pendlebury, was held on Monday, December 30, 1912, and a large number of subscribers and supporters availed themselves of the invitation of the Board of Governors to be present. The visitors were received by the Chairman of the House Committee, Mr. William Buckley, J.P., and the afternoon's arrangements were in the hands of the Chairman of the Board, Mr. A. Heywood; Mr. Stewart Garnett, J.P., one of the Trustees; the Chairman of the Medical Board, Dr. Christopher C. Heywood, M.R.C.P.; Mr. George Comber, J.P., and Mr. A. A. Gillies, J.P., Hon. Treasurers; Mr. John Dendy, the Hon. Secretary; and Mr. Charles Roberts, F.R.C.S., Senior Visiting Surgeon, besides many other governors.

The Dean of Manchester, Bishop Welldon, paid a welcome visit of inspection to all the wards, in each a member of the governing body and hon. staff taking the pleasant task of giving out the Christmas presents to the young patients. The attendance in each ward during the Christmas celebrations of a member of the board and a member of the hon. staff, who jointly attach themselves during the afternoon to an individual ward in which they may be interested, and devote themselves whole-heartedly to the successful rendering of the Christmas spirit throughout the hospital, is a pleasing feature of the annual "Tree-Day" at Pendlebury. The six huge Christmas trees, each 18 feet high, were lit by 216 coloured electric lamps, a gift from a firm of electrical engineers in the city. The Borchardt Ward Christmas tree, dressed by Sister Blunt and her nurses, was a beautiful representation of "Babies in Poppyland." The Victoria Ward, with thirty beds, maintained by the Ladies' Fund District Committees, in charge of Sister Staley, contained a Christmas tree entirely Japanese in character. Wrigley Ward reflected great credit on Sister Foulkes, with its large tree holding excellent reproductions of all the nursery rhymes dear to the hearts of children, and the same could be said of the Christmas tree in the Heywood Ward, dressed by Sister Stower and her nurses to represent "Fairyland." A novel scheme of decoration had been carried out in the Holden Ward (founded by the Holden Trustees) by Sister Gledhill and her nurses to represent the British Isles. In Liebert Ward Sister Ashe and her nurses produced excellent results with a Christmas tree to represent "Winter."

Mr. Arthur Heywood, the Chairman of the Board, had arranged for the Holyrood Choir to attend and sing carols during the afternoon, and these were much appreciated by all present. Still further joys for the patients at this, the second largest children's hospital in the kingdom, followed last Monday, when the New Year's tea-party, given by the Chairman of the House Committee, Mr. William Buckley, J.P., took place, and which included, in addition, a cinematograph entertainment in each of the six wards.

At the convalescent branch of the hospital at St. Anne's-on-the-Sea Miss Stevens, the matron, who has had charge of this branch for over sixteen years, held a "Tree-Day" and Christmas party on December 30 for the convalescents from Pendlebury.

